# Analysis of fecal microbiome and metabolome changes in goats with pregnant toxemia

**DOI:** 10.1186/s12917-023-03849-0

**Published:** 2024-01-03

**Authors:** Bingyan Jin, Ruoqian Wang, Jiada Hu, Yan Wang, Panpan Cheng, Jiancong Zhang, Jiahui Zhang, Gang Xue, Yan Zhu, Yunhai Zhang, Fugui Fang, Ya Liu, Yunsheng Li

**Affiliations:** Anhui Provincial Key Laboratory of Local Livestock Poultry, Genetical Resource Conservation and Breeding, College of Animal Science and Technology, Anhui Agriculture University, 130 Changjiang West Road, 230036 Hefei, Anhui China

**Keywords:** Pregnancy toxemia, Microbiome, Metabolomics, 16S rRNA

## Abstract

**Background:**

Pregnancy toxemia is a common disease, which occurs in older does that are pregnant with multiple lambs in the third trimester. Most of the sick goats die within a few days, which can seriously impact the economic benefits of goat breeding enterprises. The disease is believed to be caused by malnutrition, stress, and other factors, that lead to the disorder of lipid metabolism, resulting in increased ketone content, ketosis, ketonuria, and neurological symptoms. However, the changes in gut microbes and their metabolism in this disease are still unclear. The objective of this experiment was to evaluate the effect of toxemia of pregnancy on the fecal microbiome and metabolomics of does.

**Results:**

Eight pregnant does suspected of having toxemia of pregnancy (PT group) and eight healthy does during the same pregnancy (NC group) were selected. Clinical symptoms and pathological changes at necropsy were observed, and liver tissue samples were collected for pathological sections. Jugular venous blood was collected before morning feeding to detect biochemical indexes. Autopsy revealed that the liver of the pregnancy toxemia goat was enlarged and earthy yellow, and the biochemical results showed that the serum levels of aspartate aminotransferase (AST) and β-hydroxybutyric acid (B-HB) in the PT group were significantly increased, while calcium (Ca) levels were significantly reduced. Sections showed extensive vacuoles in liver tissue sections. The microbiome analysis found that the richness and diversity of the PT microbiota were significantly reduced. Metabolomic analysis showed that 125 differential metabolites were screened in positive ion mode and enriched in 12 metabolic pathways. In negative ion mode, 100 differential metabolites were screened and enriched in 7 metabolic pathways.

**Conclusions:**

Evidence has shown that the occurrence of pregnancy toxemia is related to gut microbiota, and further studies are needed to investigate its pathogenesis and provide research basis for future preventive measures of this disease.

**Supplementary Information:**

The online version contains supplementary material available at 10.1186/s12917-023-03849-0.

## Background

Pregnancy toxemia is a nutritional metabolic disease caused by malnutrition and feeding management. It is more commonly observed in late-gestational does raised in intensive enclosures, particularly elderly does carrying twins or multiple births [1, 2]. The disease is characterized by hyperketonemia, hypocalcemia, hepatic steatosis, and metabolic acidosis [3, 4], along with systemic symptoms such as depression, vision loss, dyskinesia, and head and neck scoliosis [4]. Typically, the disease occurs within two weeks, or even days, before farrowing, with an incidence of approximately 5-20%, and a mortality rate of over 80% [5, 6]. During late pregnancy, the growing fetus demands more nutrition, while the increasing fetal volume compresses the rumen volume maternal nutrient intake. This creates a serious negative energy balance state, as higher nutrient requirements conflict with lower nutrient intake [7, 8]. Xue et al. successfully replicated a pathological model of gestational malnutrition by restricting feeding, and found that the expression of liver genes for fatty acid oxidation and triglyceride synthesis in pregnant ewes was enhanced, while the synthesis of triglycerides in the fetal liver was increased and degradation decreased [9]. The microbiota of the rumen and colon were also altered by restricted feeding, leading to inhibition of rumen epithelial cell proliferation, and changes in colonic fermentation patterns and epithelial tissue morphology [10, 11]. Pregnancy toxemia is a metabolic disorder, and while the dynamics of the disorder still unclear, Guo et al. identified differential metabolites in the blood of goats during feeding restriction [12]. Treatment of pregnancy toxemia typically involves glucose supplementation to protect the liver, promote metabolism, correct acidosis, and symptomatic therapy. Guo et al. added glycerol and rumen protector choline chloride to the diet of pathological model goats and found that these compounds counteracted to detect the content of metabolites in the blood and urine of the model goat. Experimental results suggest that glycerol and choline chloride counteract some of the changes in metabolite concentrations due to feeding restriction [13, 14].

Gut microorganisms, primarily bacteria, play a crucial role in a variety of physiological processes in mammals, including nutrient metabolism, immune protection, homeostasis, and body development. [15]. Genton et al. report that malnutrition reduces the barrier function of the intestine and changes the structure and composition of the intestinal flora [16]. Xue et al. found that restricted feeding also led to changes in the microbiota of the rumen and colon, as well as their fermentation patterns [10, 11]. However, there has been little research on changes in the gut microbiota and metabolism of spontaneous does with Pregnancy toxemia.

In this study, we hypothesized that fecal microbiome and metabolome were altered in goats with pregnant toxemia. Fecal samples from naturally occurring does were collected and analyzed using 16S rRNA and LC-MS to explore changes in the fecal microbiome and metabolome. These finding may provide a basis for future research into the prevention and treatment of eclampsia in goats.

## Results

### Diagnosis of pregnancy toxemia in goats

**Fig. 1:**
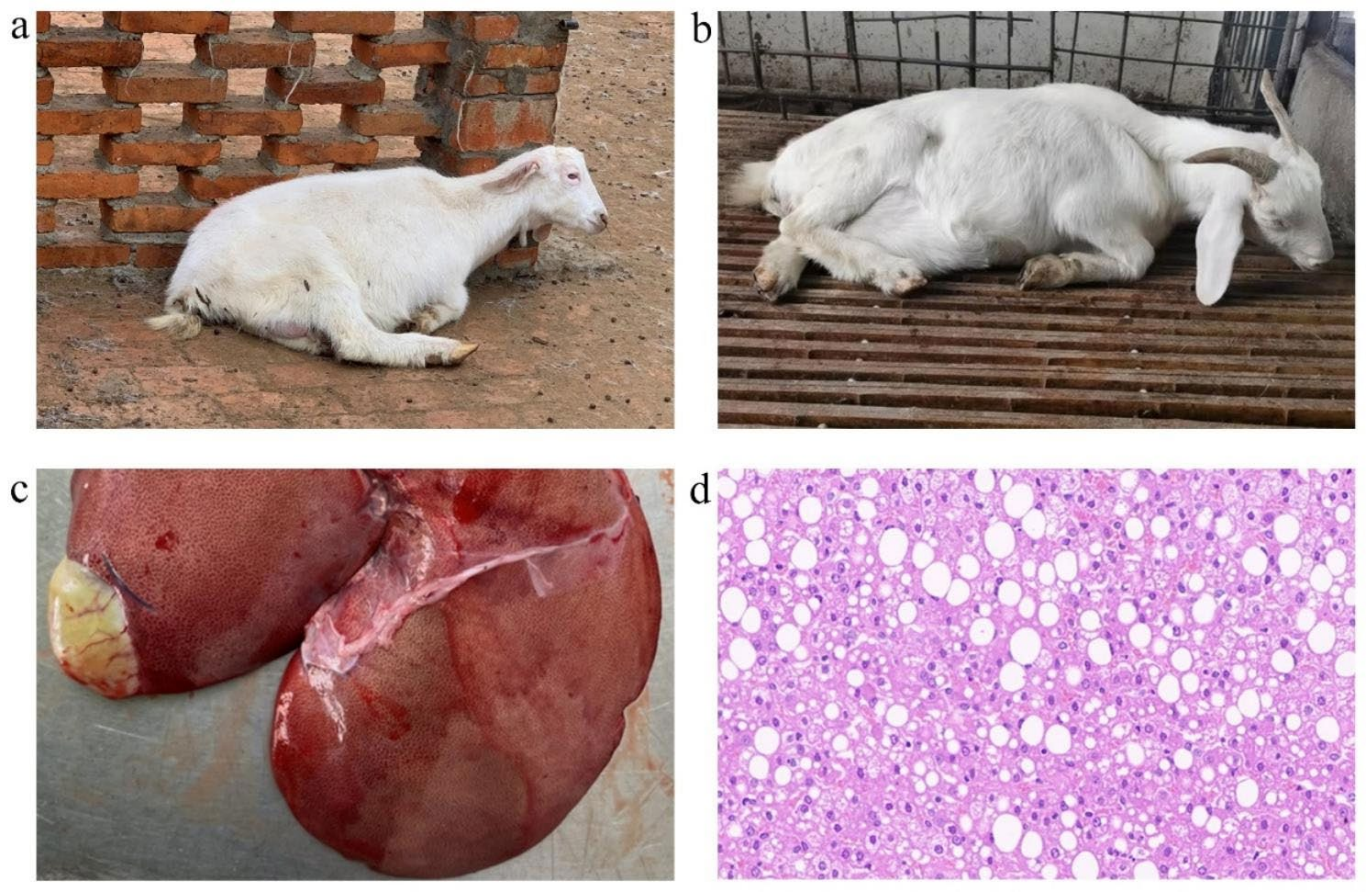
**Clinical symptoms and pathological changes of liver in PT group does.** (**a-b**) Clinical symptoms of does in the PT group. (**c**) Visual examination of the liver. (**d**) Numerous vacuoles in the liver tissue of does in the PT group

Does with suspected pregnancy toxemia exhibited clinical symptoms such as depressed spirits, loss of appetite, and an inability to lie on the ground (Fig. 1a), as well as tachypnea. Some diseased goats developed severe mental symptoms such as a head and neck tilt to the ventral costal region (Fig. 1b), and most of the sick goats died within 1–3 days. Autopsies showed that the livers of the sick goats were enlarged and earthy yellow, with rounded edges, a soft texture, reddish and yellow texture on the surface, and an enlarged gallbladder (Fig. 1c). Liver tissue sections showed numerous vacuoles in the cytoplasm of the cells, with variably sized vacuoles in the cytoplasm pushing the nuclei to one side (Fig. 1d).

The blood biochemical results (Table 1) showed that the level of AST in goats with suspected pregnancy toxemia was significantly higher than that in the control group (*P* < 0.05), the level of Ca ion was significantly lower than that of the control group (*P* < 0.01), the level of B-HB was significantly higher than that in the control group (*P* < 0.01), and the levels of the remaining 16 biochemical indexes were not significantly different from the control group (*P* > 0.05).

**Table 1:** Test results of blood biochemical indicators

Item	Groups		*P*-value
	PT(n = 8)	NC(n = 8)	
ALT (U/L)	28.08 ± 3.96	23.96 ± 1.67	0.247
AST (U/L)	183.46 ± 31.97	112.36 ± 4.51	0.017
ALP (U/L)	400.02 ± 225.37	158.38 ± 22.34	0.196
r-GGT (U/L)	45.58 ± 7.99	44.94 ± 2.73	0.929
TP (g/L)	60.08 ± 1.54	66.14 ± 2.40	0.095
ALB (g/L)	21.14 ± 0.41	23.09 ± 0.74	0.078
GLB (g/L)	38.94 ± 1.15	43.05 ± 1.90	0.142
A/G(%)	0.52 ± 0.020	0.52 ± 0.025	0.891
TBIL-V(µmol/L)	6.15 ± 0.79	5.07 ± 0.335	0.177
UREA(mmol/L)	6.12 ± 0.32	6.47 ± 0.37	0.533
GLU(mmol/L)	0.99 ± 0.48	1.38 ± 0.32	0.498
Ca(mmol/L)	1.78 ± 0.03	2.08 ± 0.02	0.000
P(mmol/L)	2.45 ± 0.19	2.24 ± 0.15	0.400
TC(mmol/L)	2.29 ± 0.40	3.01 ± 0.35	0.211
TG(mmol/L)	0.22 ± 0.04	0.37 ± 0.55	0.094
CK (U/L)	379.24 ± 156.77	189.93 ± 11.24	0.147
LDH (U/L)	281.18 ± 36.82	278.89 ± 15.40	0.948
FUN(µmol/L)	339.98 ± 139.76	198.30 ± 3.94	0.215
B-HB(mmol/L)	4.90 ± 0.59	0.39 ± 0.07	0.000

ALT: Alanine aminotransferase; AST: Aspartate aminotransferase; ALP: Alkaline phosphatase; GGT: Glutamyl transpeptidase; TP: Total protein; ALB: Albumin; GLB: Globulin; A/G: Albumin /globulin; TBIL-V: Total Bilirubin; GLU: Glucose; Ca: Calcium; P: Phosphorus; TC: Total cholesterol; TG: Triglyceride; CK: Creatine kinase; LDH: Lactate dehydrogenase; FUN: Fructosamine; B-HB: β-hydroxybutyric acid

### Overall structure of fecal bacterial communities

**Fig. 2:**
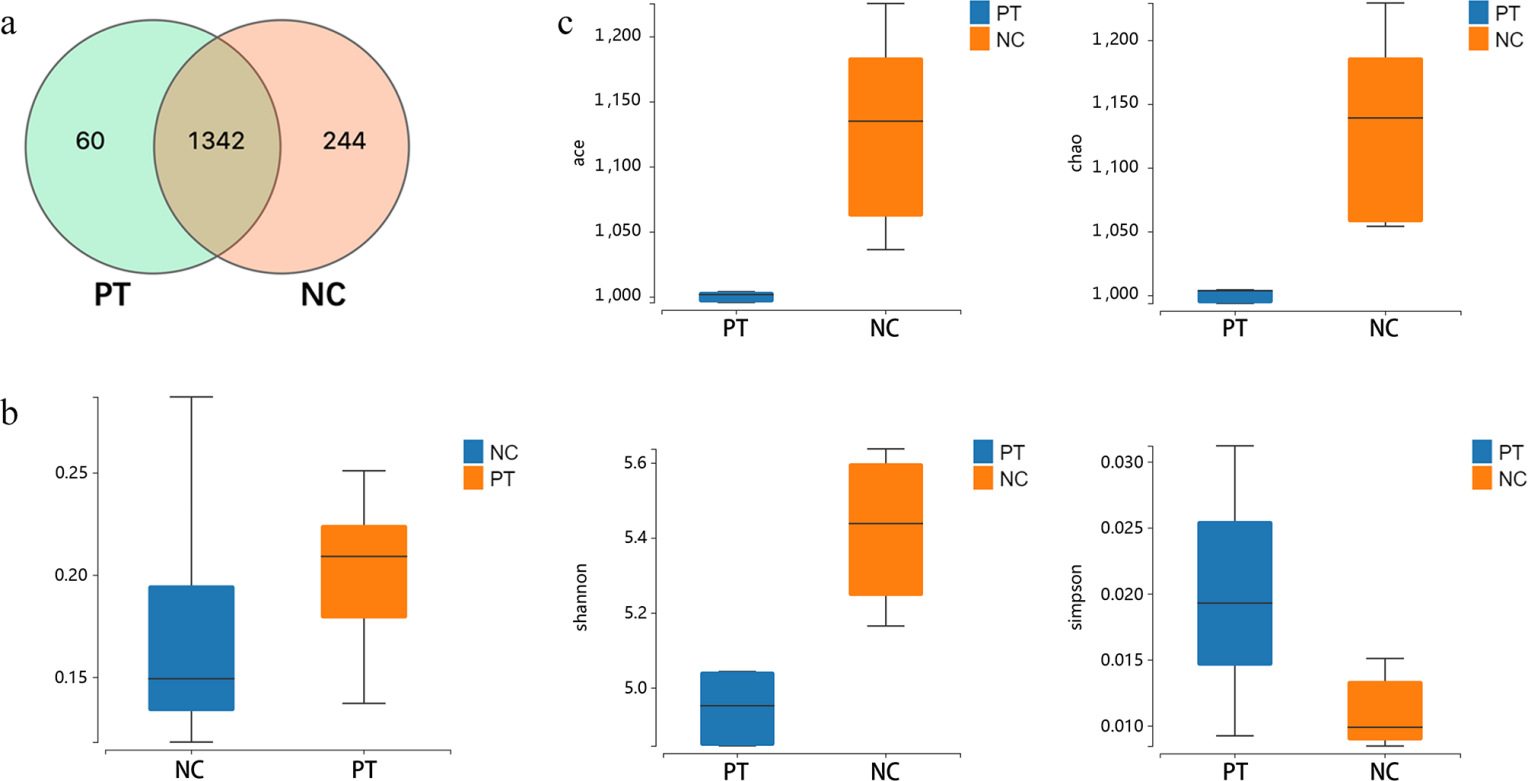
**Effects of pregnancy toxemia does on fecal microbial community diversity.** (**a**)Venn diagrams of OTUs in two groups. (**b**)β intergroup diversity analysis. (**c)**Alpha diversity index between-group difference box plot

In the PT groups, 1402 OTUs were obtained,while the NC group 1586 OTUs.Combined,there were 1342 OTUs in both groups. Figure 2a shows that the NC group had more unique OTUs than the PT group. Beta diversity analysis revealed significant differences in microbial structural composition between the two groups (Fig. 2b). Meanwhile, Alpha diversity analysis showed that the ACE, Shannon, and Chao1 indices of the PT group were significantly lower than those of the NC group (*P* < 0.05), while the Simpson index was significantly higher than that of the NC group (*P* < 0.05) (Fig. 2c). These results indicate significant differences in the microbial composition, diversity, and abundance of goat feces between the PT and NC groups. Specifically, the PT group had a reduced microbial diversity and abundance.

### Analysis on composition and difference of microbiota

**Fig. 3:**
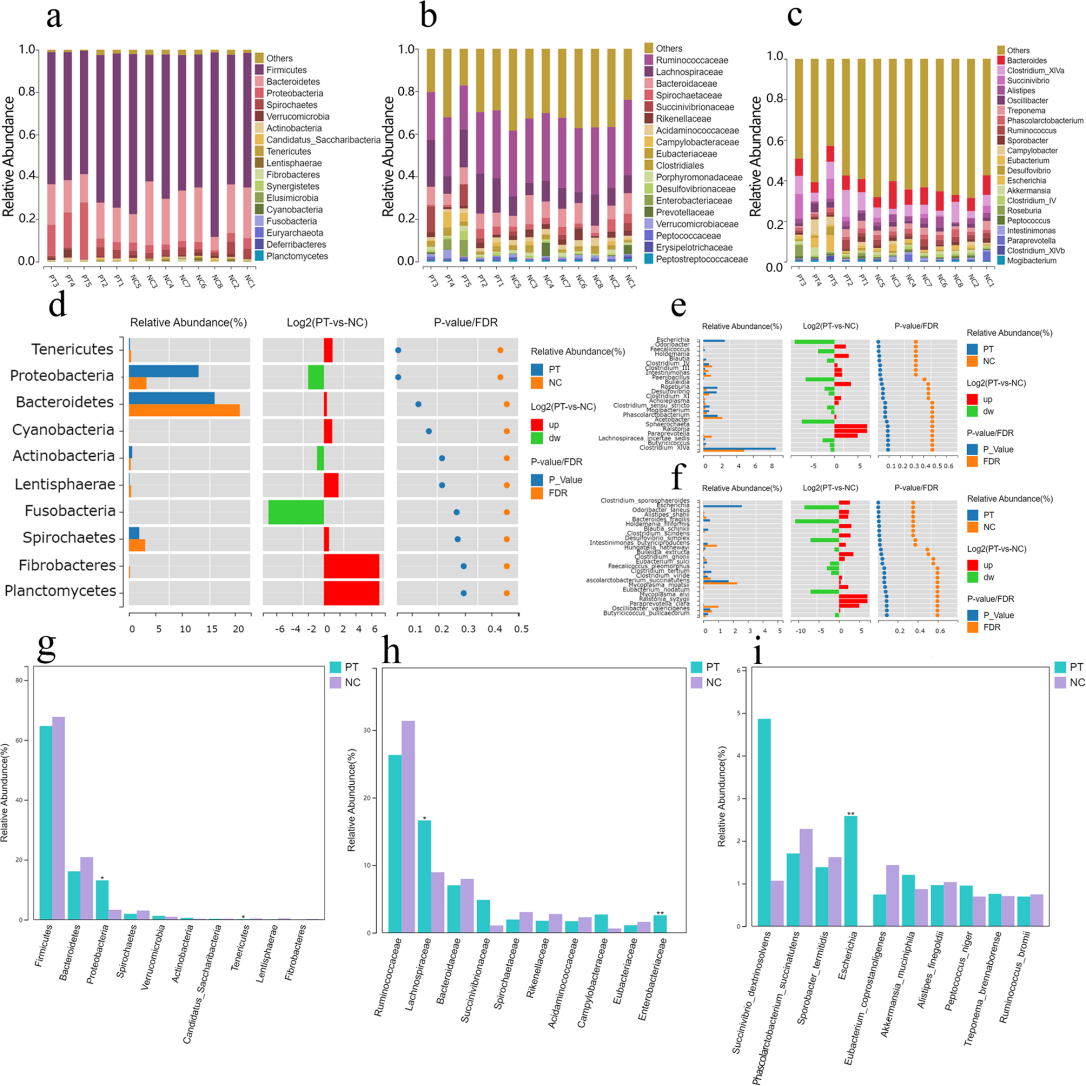
**Composition and difference analysis of fecal microbial communities in PT and NC groups.** (**a-c**) Species composition analysis at the phylum, family and genera levels between PT and NC groups. (**d-f**) Analysis of species differences at the bacterial phylum, genus and species levels between PT and NC groups. (**g-i**) Analysis of key species differences at the bacterial phylum, family, and species levels between PT and NC groups. All the results are shown as the mean ± SEM; * *p* < 0.05, ** *p* < 0.01

Figure 3a and c shows the species composition of fecal microorganisms in the PT group and NC group. At the phylum level (Fig. 3a), the phylums with relative abundances greater than 10% in the PT group were Firmicutes (64.55%), Bacteroidetes (16.10%) and Proteobacteria (13.37%), and the phylum with relative abundances greater than 10% in the NC group were Firmicutes (68.34%) and Bacteroidetes (20.43%). At the family level (Fig. 3b), the families with relative abundances greater than 10% in the PT group were Ruminococcaceae (26.23%) and Lachnospiraceae (16.62%), and the families with relative divisions greater than 10% in the NC group were Ruminococcaceae (31.37%). At the genus level (Fig. 3c), the genera with relative abundances greater than 5% in the PT group were Clostridium_XlVa, (8.69%) and Bacteroides (7.03%), while genera with relative abundances greater than 5% in the NC group were Bacteroides (7.81%) and Clostridium_XlVa (5.03%).

The analysis of species difference in fecal microorganisms between the PT group and NC group is presented in Fig. 3d-f. At the phylum level (Fig. 3d), the relative abundance of Tenericutes was significantly lower in the PT group than in the NC group (*P* < 0.05), while the relative abundance of Proteobacteria was significantly higher in the PT group than in the NC group (*P* < 0.05). At the genus level (Fig. 3e), the relative abundance of Escherichia, Roseburia, Desulfovibrio, and Paenibacillus was significantly higher in the PT group than in the NC group (*P* < 0.05), While the relative abundance of Clostridium_IV, Intestinimonas, and Holdemania was significantly lower in the PT group than in the NC group(*P* < 0.05). At the species level (Fig. 3f), the relative abundance of Holdemania_filiformis, Desulfovibrio_simplex and Escherichia in the PT group was significantly higher than t in the NC group (*P* < 0.05). While the relative abundance of Odoribacter_laneus, Clostridium_scindens and Intestinimonas_butyriciproducens was significantly lower than in the NC group (*P* < 0.05).

To further investigate the key species differences between fecal microorganisms in the PT group and the NC group, the top 10 species with average relative abundance were selected for analysis. At the phylum level (Fig. 3g), the relative abundance of Proteobacteria was significantly higher in the PT group than in the NC group (*P* < 0.05), while the relative abundance of Tenericutes was significantly lower in the PT group than in the NC group (*P* < 0.05). At the family level (Fig. 3h), the relative abundance of Lachnospiraceae was significantly higher in the PT group than in the NC group (*P* < 0.05), while the relative abundance of Enterobacteriaceae was significantly higher in the PT group than in the NC group (*P* < 0.01). At the species level (Fig. 3i), the relative abundance of Escherichia was significantly higher in the PT group than in the NC group (*P* < 0.01).

### Composition change and functional difference analysis of microbiota

**Fig. 4:**
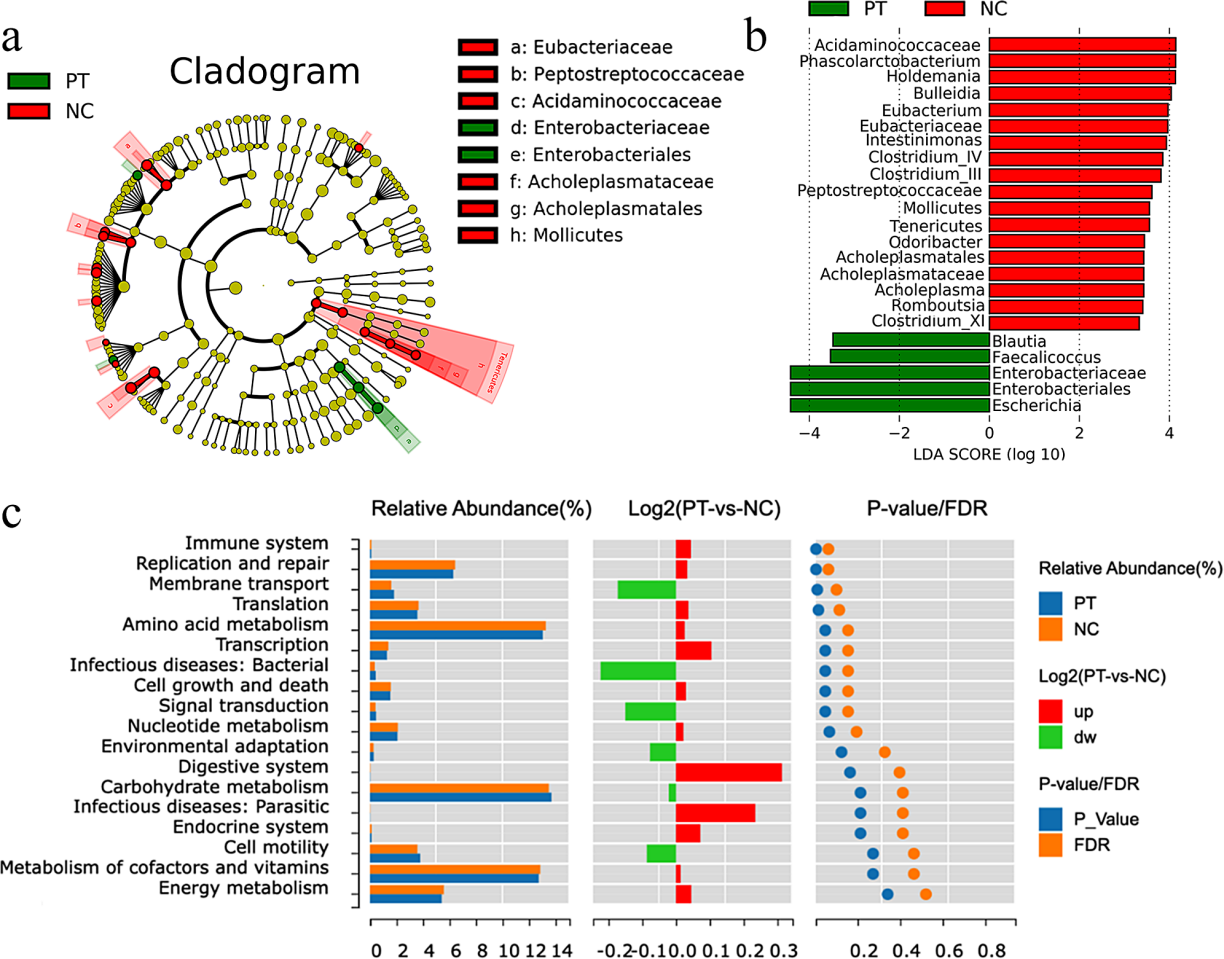
**Analysis of functional differences in fecal microbial communities.** (**a**)LEfSe identified the taxa with the greatest difference between the PT and NC groups, a claria map obtained from LEfSe analysis of the 16 S sequence. (**b**)The LDA score was positive (red) for the PT-enriched taxa and positive (green) for the NC-enriched taxa, showing only the taxa of LDA > 2. (**c**)Comparative analysis chart of functional differences

As shown in Fig. 4a and b, the relative abundance of Blautia, Faecalicoccus, Enterobacteriaceae, Enterobacteriales, and Escherichia in the PT group were significantly higher than in the NC group (*P* < 0.05). Conversely, Acidaminococcaceae, Phascolarctobacterium, Holdemania, Intestinimonas, Clostridium_IV, Clostridium_III, Odoribacter, and Acholeplasma had significantly lower than relative abundance in the PT group compared to the NC group (*P* < 0.05).

As shown in Fig. 4c, pregnancy toxemia goat fecal microorganisms are mainly involved in carbonic acid metabolism, cofactor and vitamin metabolism, terpene and polyketone metabolism, amino acid metabolism and other functions. The functions of fecal microbial amino acid metabolism, immune system, replication and repair, cell growth and death, and translation in the PT group were significantly lower than those in the NC group (*P* < 0.05), and the functions of membrane transport, transcription, infectious bacteria and signaling were significantly higher than those in the NC group (*P* < 0.05).

### Classification of metabolites and functional notes

**Fig. 5:**
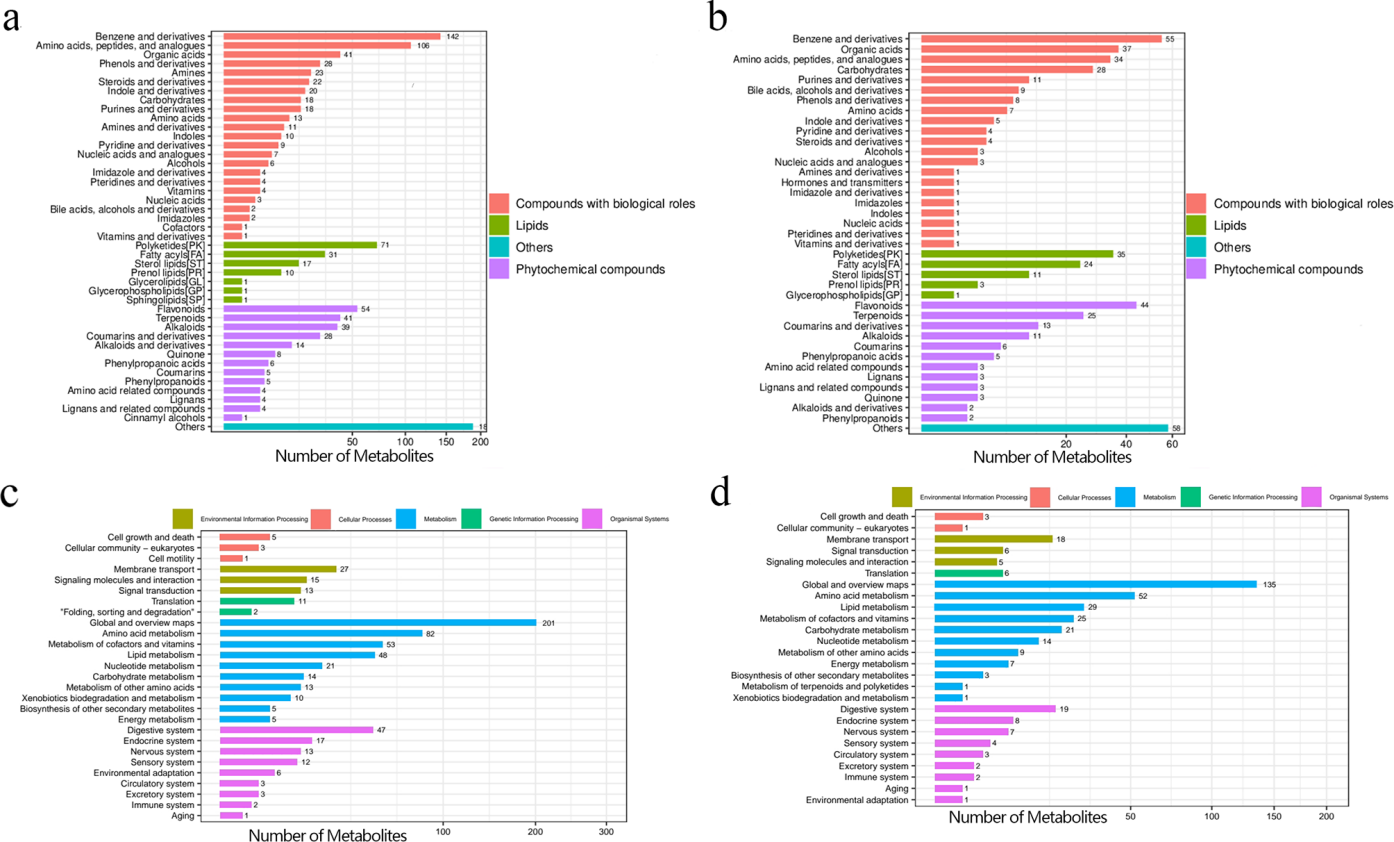
**Analysis of fecal metabolites in PT and NC groups in positive and negative ion mode.** (**a**) Bar chart of metabolite classification in positive ion mode. (**b**) Bar chart of metabolite classification in negative ion mode; (**c**) KEGG function annotation bar chart in positive ion mode; (**d**) KEGG function annotation bar chart in negative ion mode

Quality control analysis indicates that the high-quality ion number meets the requirements and the model predictions are accurate (Additional file 1). In positive and negative ion modes, metabolites are mainly divided into compounds with biological effects, lipids, plant compounds, and others (Fig. 5a and b). Compounds with biological effects mainly included benzene and its derivatives, amino acids and peptide derivatives and organic acids, while lipids comprised polyketone compounds, fatty acyls, sterol lipids and pregnenol lipids. Plant compounds mainly include flavonoids and terpenoids. These metabolites were associated with functions such as environmental information processing, metabolism, and organic systems (Fig. 5c and d). Metabolites were primarily involved in environmental information processing, such as membrane transport and signaling molecules and their interactions. They also participated in metabolic processes such as amino acid and vitamin metabolism, and played a role in organic systems like the digestive, nervous and immune.

### Analysis of differential metabolites

**Fig. 6:**
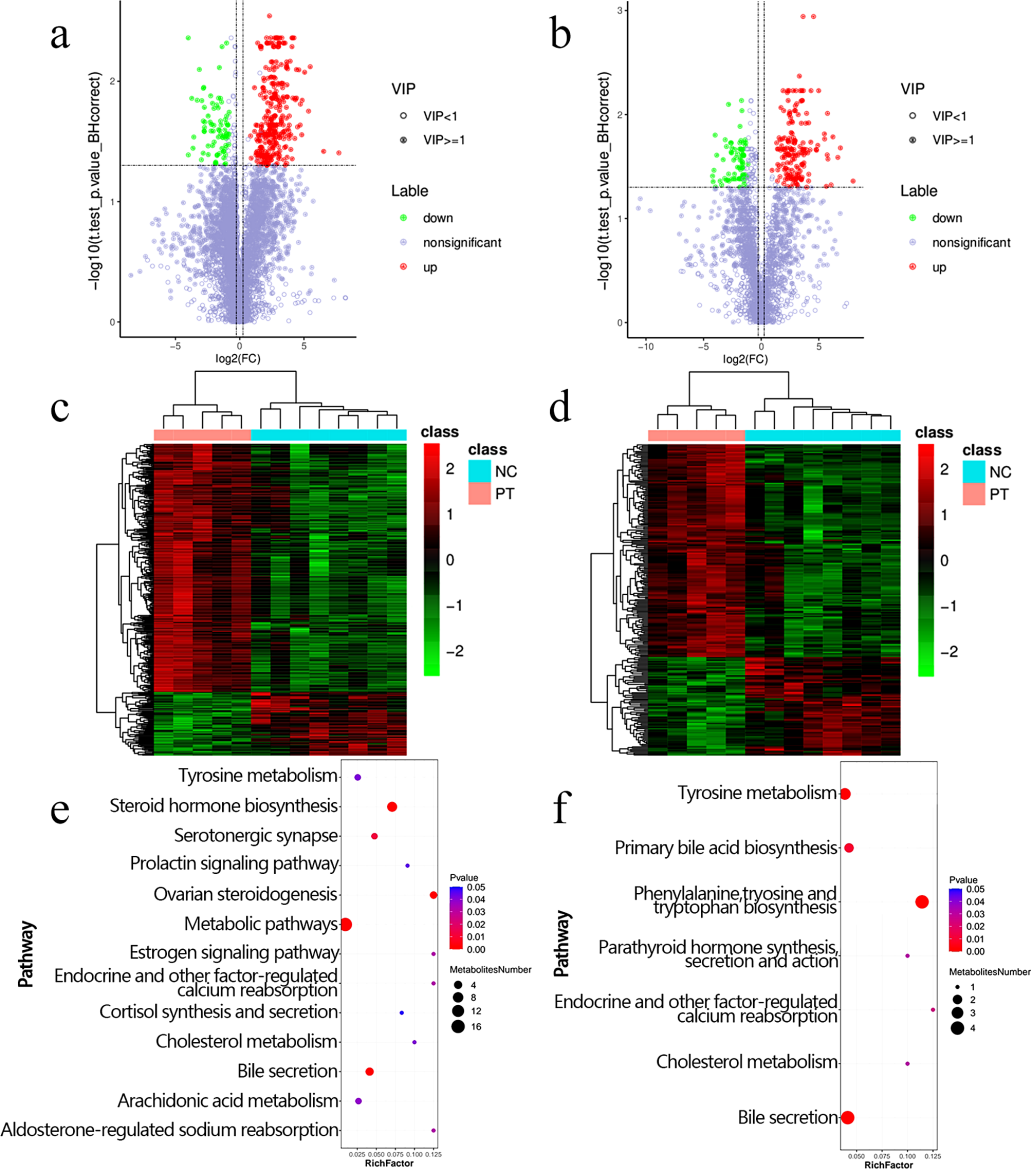
**Analysis of differences in fecal metabolites between the PT group and the NC group.** (**a**)Volcano map of metabolites in PT and NC groups in positive ion mode. (**b**)Volcano map of metabolites in PT group and NC group in negative ion mode. (**c**) Clustering of differential metabolites between PT and NC groups in positive ion mode. (**d**)Cluster analysis of differential metabolites in PT group and NC group in negative ion mode. (**e)** Bubble plot of metabolic pathway enrichment analysis of PT group and NC group in positive ion mode. (**f**) Bubble plot of metabolic pathway enrichment analysis of PT group and NC group in negative ion mode

The results of the volcano plot and cluster analysis showed that the fecal metabolites of the PT group and the NC group were quite different. In positive ion mode, the total number of differential metabolites was 125, of which 89 were upregulated and 36 were down-regulated. In negative ion mode, the total number of differential metabolites was 100, of which 51 were upregulated and 49 were downregulated (Fig. 6a-d, Additional file 2). Additional file 3 lists the top 10 up-regulated and down-regulated differential metabolites in positive and negative ion modes. The bubble plot shows the degree of enrichment of fecal differential metabolites in different metabolic pathways, as well as the number of differential metabolites enriched in each pathway. In positive ion mode, 12 metabolic pathways were enriched and 13 differential metabolites were significantly enriched. In negative ion mode, 7 metabolic pathways were enriched and 12 differential metabolites were significantly enriched (Fig. 6e-f, Additional file 4). Additional file 5 lists the metabolic pathways in which differential metabolites are significantly enriched in positive and negative ion modes.

In positive ion mode, the PT group showed upregulated levels of metabolites such as cortisol, dehydroepiandrosterone, β-estradiol, and testosterone in steroid hormone biosynthesis (Additional file 6). Additionally, in bile secretion (Additional file 7), the levels of metabolites such as thromboxane b2, cortisol and deoxycholate were upregulated in the PT group. In negative ion mode, the levels of metabolites such as chenodeoxycholate, deoxycholic acid and lithocholic acid were upregulated in bile secretion, while the content of glycocholate was down-regulated (Additional file 8). Moreover, in the metabolic pathways of phenylalanine, tyrosine and tryptophan biosynthesis, the level of chenodeoxycholate in fecal metabolites of the PT group was up-regulates, while the level of glycocholate was down-regulated (Additional file 9).

### Comprehensive analysis of microbiome and metabolomics

**Fig. 7:**
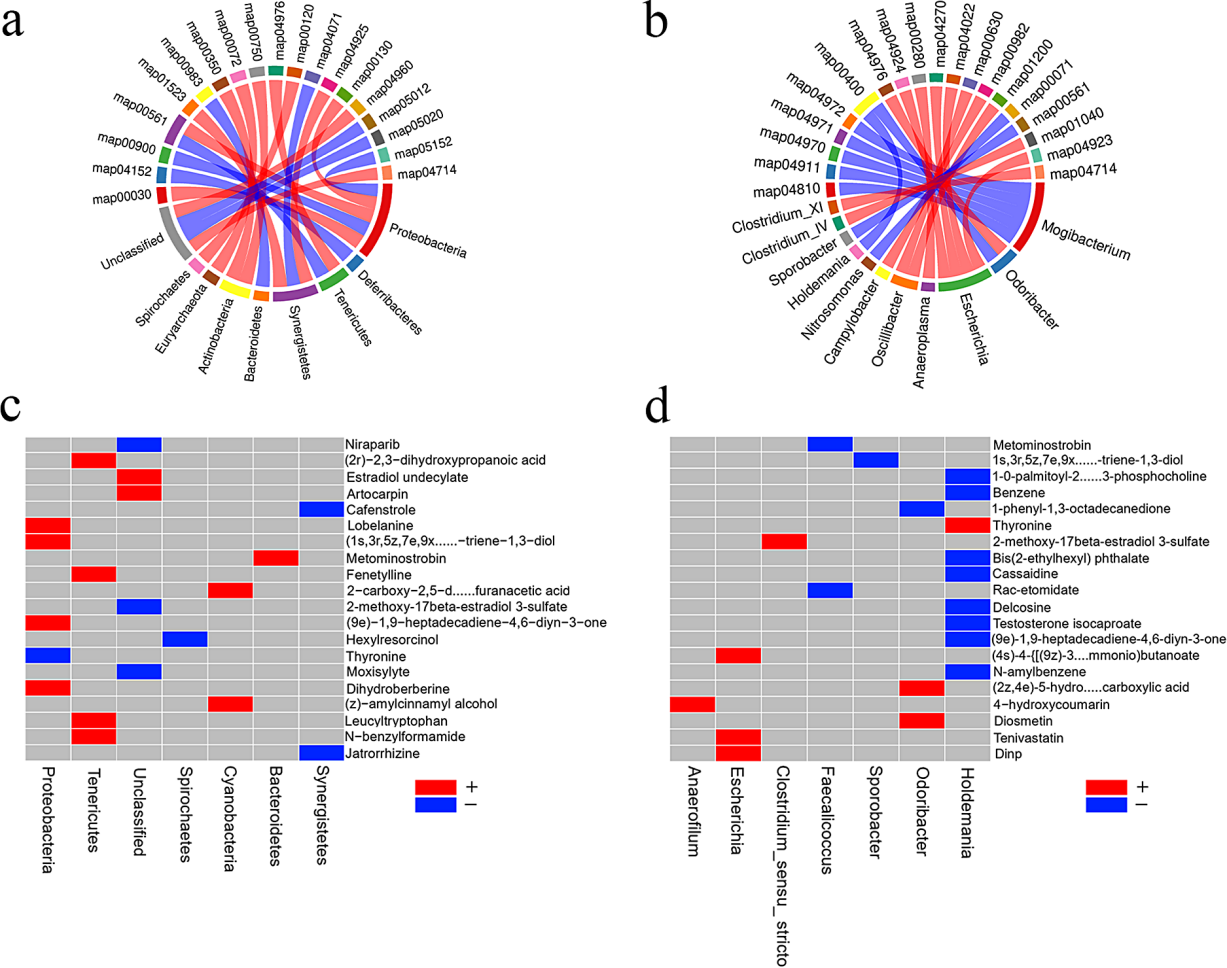
**Comprehensive Analyses of the Microbiome and Metabolome.** (**a**)Chord diagrams of metabolic pathways and microbial community correlations at the phylum level. (**b**) Metabolic pathways and microbial community correlation and chord phylum levels at the genus level. (**c**)Phylum-level differential metabolites correlated with microbial taxa heatmap. (**d**)Heat map of correlation between horizontal difference metabolites and microbial groups

The correlation and chord diagram between differential metabolites and microflora showed that, at the phylum level, the metabolic pathways negatively correlated with Proteus phylum abundance included pentose phosphate metabolism, glycerol ester metabolism, ubiquinone biosynthesis, and so on, while terpenoid metabolic pathways positively correlated with Proteus phylum abundance. Anti-folic acid and aldosterone synthesis were negatively correlated with the abundance of interoxophiles, while sphingolipid signaling pathway was positively correlated with them. The tyrosine and vitamin B6 metabolism were the metabolic pathways negatively correlated with Actinomyces phylum abundance. Bile metabolism was negatively correlated with the abundance of Firmicutes, while glycerol ester metabolism was positively correlated. The primary bile acid biosynthesis pathway was negatively correlated with spirochete abundance (Fig. 7a). At the genus level, metabolic pathways negatively correlated with Escherichia abundance included renin secretion, vascular smooth muscle contraction, cGMP-PKG signaling pathway, and regulation of lipolysis. Carbon metabolism and glyoxylate and dicarboxylic acid metabolism were negatively correlated with spirochete abundance. Phenylalanine, tyrosine, and tryptophan anabolism were positively correlated with odor bacillus abundance and Haldeman’s abundance, while bile secretion metabolic pathways were negatively correlated with odor bacillus abundance. Thermogenesis and unsaturated fatty acid biosynthesis in metabolic pathways negatively correlated with clostridium abundance. Amino acid metabolic pathways such as valine, leucine, and isoleucine were negatively correlated with the abundance of anaerobic protozoa (Fig. 7b).

The heatmap of correlation between differential metabolites and microflora showed that at the phylum level, N-benzyltrioxane ketone and dihydrokabasine were metabolites positively correlated with Tenericutes, while formamide, phenethyltheophylline, leucine, tryptophan, heptadecenedione, trienediol, and thyronine were negatively correlated with Tenericutes. Amyl cinnamyl alcohol and carboxy furosine alcohol were metabolites positively correlated with cyanophyta. Phenylsulfone azole and jatrorrhizine were metabolites negatively correlated with mycophenolate mofetil. Hexylresorcinol was negatively correlated with spirochetes (Fig. 7c). At the genus level, n-amylbenzene, diethyl hexyl phthalate, delphinine, and testosterone isoate were metabolites negatively correlated with Haldemanella, while thyronine was positively correlated with them. Metabolites of ammonium butyrate, diisononyl phthalate, and tenivastatin were positively correlated with Escherichia. Metabolites positively associated with the genus Odorbacillus were diosmin and hydrogenated dicarboxylic acids, with diphenyl octadecone negatively associated. Metabolites such as etomidate and Metominol were negatively correlated with coprococcus (Fig. 7d).

## Discussion

### Changes in the fecal microbiome of pregnancy toxemia goats

In this study, through the OUT Wayne diagram and α diversity analysis, it was found that the richness and diversity of fecal microorganisms in the PT group were significantly lower than those in the NC group. That is, the richness and diversity of fecal microorganisms in pregnancy toxemia goats were significantly reduced, which was similar to the change in the intestinal microbial composition of pregnancy toxemia ewes observed in Xue et al. [11].

Microbiome analysis revealed a significant increase in the relative abundance of Proteobacteria and Escherichia in the feces of pregnancy toxemia goats, along with a significant decrease in the relative abundance of Tenericutes and Clostridium. Previous studies have linked the increase in Proteobacteria abundance in the intestine with various metabolic diseases, including type 2 diabetes mellitus [17], obesity [18] and nonalcoholic steatohepatitis [18]. Moreover, elevated Proteobacteria abundance has been observed in many intestinal inflammatory diseases. Tenericutes are primarily involved in the synthesis of host intestinal carbohydrates and amino acid metabolism [19]. In this study, the abundance of Proteobacteria in the feces of pregnancy toxemia goats increased significantly, which may cause inflammatory response in the intestinal tract of ewes [11], and the relative abundance of Tenericutes was significantly reduced, which may be related to the weakening of digestion and absorption function of the gastrointestinal tract of sick goat, aggravated the negative energy balance state of does, and promoted the occurrence of pregnancy toxemia in goats.

The most common species in the genus Escherichia coli is Escherichia coli. Enteropathogenic E. coli causes host diarrhea through mechanisms such as increased intestinal permeability and inflammatory response [20]. In this study, the fecal samples of pregnancy toxemia goats were all soft and unformed, indicating mild diarrhea in the intestines of sick goats, which may be related to the increased abundance of Escherichia species. Clostridium bacteria can break down dietary fiber to produce organic acids such as butyric acid [21], which is the main source of energy for colonic epithelial cells. This strengthens the integrity of the intestinal epithelial barrier and inhibits the inflammatory response [22]. In this study, the abundance of Clostridium in the feces of pregnancy toxemia goats was significantly reduced, which may lead to a decrease in butyrate content in the colon of diseased goats. This, in turn, damaged the colonic epithelium and caused an inflammatory response. The results of this study are similar to those of the previous dominant flora study, which found that the colonic flora changed due to the diet restriction mold test belonging to the order Clostridium and Clostridiales [11].

### Effects of pregnancy toxemia on steroid hormone and fatty acid metabolism

In this study, the content of testosterone, estradiol and cortisol in the fecal metabolites of pregnancy toxemia goats was significantly increased, indicating involvement in the steroid hormone bioanabolic pathway. Steroid hormones include sex hormones, adrenal corticosteroids and 1,25-dihydroxyvitamin D3, which have anti-inflammatory and anti-allergic effects and are involved in the body’s metabolic processes. When the body’s calcium content decreases, it induces an increase in the secretion of 1,25-dihydroxyvitamin D3, mobilizes bone calcium into the blood, promotes the absorption of calcium and phosphorus in the intestine, and increases the calcium and phosphorus content in the blood. Consistent with the findings of Andrew et al., cortisol levels in pre-eclampemic goat were also elevated in this experiment [23]. Researchers believe that the body may be in a state of hypoglycemia for a long time due to the reduction of liver damage metabolism, which promotes cortisol secretion, and thereby alleviating the negative energy balance in the body [23, 24].

Fatty acids are the main components of fat, and the content of thromboxane B2 and 11-dehydrothromboxane B2 in the fecal metabolites of pregnancy toxemia goats in this study was significantly increased, involving the arachidonic acid metabolic pathway. Arachidonic acid is an important essential fatty acid and a direct precursor to various bioactive substances such as prostaglandins, thromboxanes, and leukotrienes, playing a crucial role in the body’s immune and inflammatory responses [25]. Studies have shown that rheumatoid arthritis can increase the expression of epoxidase and lipoxidase, significantly increasing the content of arachidonic acid and its metabolites in plasma [26]. Furthermore, the arachidonic acid metabolic pathway is vital pathway for inducing inflammatory responses. The increased content of thromboxane B2 and 11-dehydrothromboxane B2 in fecal metabolites of pregnancy toxemia goats can cause an increased in arachidonic acid content, which may be related to intestinal inflammation. Xue et al. found that the content of fatty acids and triglycerides in the blood of sick goats increased significantly through restricted feeding modeling [12]. The content of blood fatty acids and triglycerides can reflect the degree of negative energy balance in the body [27]. In addition, when the body is in a state of negative energy balance, fatty acids can be converted into ketone bodies in the liver. High concentrations of ketone bodies penetrate the blood-brain barrier and enter the brain tissue, affecting the nervous system and leading to neurological symptoms, such as erratic movements and circular motion. In this study, there were fewer fatty acid-related metabolites in fecal metabolites in pregnancy toxemia goats, possibly due to differences between fecal metabolites and blood metabolites, as well as fecal metabolites being influenced by gut microbial metabolic function. Therefore, there were differences in the differential metabolites of the restricted feeding goat pregnancy toxemia model and the naturally occurring goat pregnancy toxemia model.

### Effects of pregnancy toxemia on amino acid metabolism

Amino acids are essential nutrients for the body. When animals are starved, the body metabolizes amino acids from muscle tissue through the gluconeogenesis pathway [28]. Previous studies have shown that pregnancy can exacerbate hypoaminoacidemia in starvation [29]. In this study, the contents of phenylethylene glycol, 3-dehydroshikimic acid, shikimic acid and 3-hydroxybenzoic acid in fecal metabolites of the PT group were significantly reduced, involved in tyrosine metabolism and phenylalanine, tyrosine and tryptophan biosynthesis and other metabolic pathways, which was similar to the results of previous studies of down-regulation of metabolites related to amino acid metabolism in the blood of pregnant toxemia ewes [12]. Amino acids can be divided into ketogenic amino acids and glycogenic amino acids according to different metabolic pathways. All glycogenic amino acids in this study were reduced, indicating that glycogenic amino acids may enter the tricarboxylic acid cycle as substrates or participate in the gluconeogenesis pathway to produce glucose.

### Effects of pregnancy toxemia on bile acid metabolism and calcium reabsorption

Bile acids are products of cholesterol metabolism and are the main components of bile. Under the influence of gut microbes, primary bile acids can be converted to secondary bile acids [30]. Studies have shown that high-fat diets, circadian rhythm disorders, drugs and hormones can change the structure of the intestinal flora, which in turn affects the bile acid content and host metabolism. In this study, there was a significant increase in the content of glycocheric acid, chenodeoxycholate, deoxycholic acid, and lithocholic acid in fecal metabolites of pregnant toxemia goats, involving bile secretion and primary bile acid biosynthetic pathways. The increased bile acid content in the feces of pregnancy toxemia goats may be related to disorders of lipid metabolism and liver damage in sick goat, and high concentrations of bile acids are toxic and may lead to inflammatory reactions and neurological symptoms, exacerbating the condition.

Calcium is an important trace element in the body. The content of calcitriol in fecal metabolites in pregnancy toxemia goats is significantly increased, involving metabolic pathways that regulate calcium reabsorption by endocrine and other factors. In this study, the serum calcium content of pregnancy toxemia goats was significantly lower than that in the control group, consistent with previous studies. In the third trimester, the need for maternal calcium increases dramatically due to rapid fetal growth and development [31]. Calcitriol can increase the absorption capacity of intestinal tissues for calcium [32], and the increase in calcitriol content in the feces of pregnancy toxemia goats may be related to the decrease in blood calcium content in sick goats and the need to improve intestinal calcium absorption.

### Comprehensive Analysis of Microbiology and Metabolomic of fecal

The relative abundance of Proteobacteria in the PT group was significantly higher than that in the NC group. The biosynthesis of pentose phosphate metabolism, glycerol ester metabolism, and ubiquinone were negatively correlated with Proteobacteria, and the metabolic pathways of terpenoids were positively correlated with Proteobacteria. This suggests that the glucose oxidative decomposition and coenzyme Q metabolism of pregnant toxemia goats might be down-regulated, while the metabolic function of polyketone compounds was up-regulated. The relative abundance of Escherichia in the PT group was significantly higher than that in the NC group. Metabolic pathways negatively correlated with the abundance of Escherichia included renin secretion, vascular smooth muscle contraction, cGMP-PKG signaling pathway, and regulation of lipolysis, suggesting that the functions of vasoconstriction and regulation of lipolysis in goats with gestational toxemia might be down-regulated. The metabolites of ammonium butyrate, diisononyl phthalate, and tenofovir were positively correlated with Escherichia. Statins could reduce blood lipid levels [33], and the abundance of Escherichia might be related to lipid metabolism.

The relative abundances of Odor bacterium, Holdemania and Clostridium. in the PT group were significantly lower than those in the NC group. The anabolism of phenylalanine, tyrosine and tryptophan was positively correlated with the abundance of Odor bacterium and Holdemania, and the bile secretion metabolic pathway was negatively correlated with the abundance of Odor bacterium. This indicates that the amino acid synthesis function of pregnancy toxemia goats may be down-regulated and the bile secretion function may be up-regulated. The metabolic pathway negatively correlated with the abundance of Clostridium has thermogenesis and biosynthesis of unsaturated fatty acids, indicating that functions such as fatty acid synthesis in goats with pregnancy toxemia may be upregulated. The relative abundance of Tenericutes in the feces of pregnancy toxemia goats was significantly reduced, and the metabolites positively correlated with the phylum were NN-Benzyltriteranone and dihydrochailine. Dihydrochailine had anti-inflammatory and lipid-lowering effects [34], and the decrease in the abundance of Tenericutes may be related to the inflammatory response and abnormal lipid metabolism in diseased goats.

## Conclusion

The microbial abundance and diversity in the feces of pregnant toxemia goats were decreased, and their fecal metabolites were found to be altered. At the genus level, the abundance of Escherichia and Roseburia in feces of the PT group was up-regulated, while the abundance of Clostridium_IV, Clostridium_III and Clostridium_IX was down-regulated. The differential metabolites identified included cortisol, thromboxane b2, deoxycholic acid and glycocholic acid. These differential metabolites were mainly enriched in the metabolic pathways of steroid hormone biosynthesis, bile secretion, phenylalanine, tyrosine and tryptophan biosynthesis, arachidonic acid metabolism and calcium reabsorption, which are regulated by endocrine and other factors.

Methods.

### Animals and sample collection

This study was conducted in the autumn of 2021, and the experimental site was located in a goat farm in Hefei, the precise geographical coordinate are 117 degrees 71 ‘east longitude and 31 degrees 95’ north latitude. A total of 16 goats with the same gestation period, including 8 goats with suspected pregnancy toxemia (PT group) and 8 healthy goats (NC group), were selected from a goat farm. All the goats used in the test were Anhui white goats, approximately one and a half years old. The test goats were uniformly managed, given free access to drinking water, and fed once a day at 7:00 and 15:00, with the detailed diet composition shown in Additional file 10. Before morning feeding, blood samples were collected from all 16 pregnant does, centrifuged at 3000 g for 10 min, and the isolated serum was aliquoted into 1.8 ml cryopreservation tubes and stored in an ultra-low temperature freezer. Stool samples were collected from the rectum of the test goats using sterile gloves, loaded into 15 ml centrifuge tubes, and stored in an ultra-low temperature freezer. For histomorphological analysis, liver tissue samples were collected through bloodletting slaughtering of unconscious does, fixed in 4% paraformaldehyde solution, and made into hematoxylin and eosin-stained sections.

### Analysis of blood biochemical indicators

The thawed serum sample was added to a centrifuge tube and loaded into an automatic biochemistry analyzer. Nineteen biochemical indexes were detected using the principle of photoelectric colorimetry.

### Microbiome analysis

DNA was extracted from fecal samples using a fecal DNA extraction kit ((D4015–02, Omega, Inc., USA). After passing the agarose gel electrophoresis test, the V3-V4 region of the fecal sample 16S rDNA was amplified using universal primers for bacteria (341F: 5’-ACTCCTACGGGAGGCAGCAGCAG-3’ and 806R: 5’-GGACTACHVGGGTWTCTAAT-3’). The PCR products were purified and a library was constructed, which was then sequenced using the Illumina Miseq platform. For detailed statistical tests performed during microbiome analysis, refer to the works of Chen et al. and Hugerth et al [35, 36].

### Metabolomic analysis

After slowly thawing the fecal samples at 4 °C, weigh 25 mg into a 1.5 mL Eppendorf tube. Add 800 µL of extraction solution (methanol:acetonitrile:water = 2:2:1, v:v:v, pre-cooled at -20 °C) and 10 µL of internal standard to the tube. Add two small steel balls and grind the mixture in a tissue grinder for 5 min at 50 Hz. Next, ultrasonicate the mixture for 10 min in a 4 °C water bath and then allow it to stand in a -20 °C refrigerator for 1 h. Centrifuge the mixture at 25,000 rpm for 15 min at 4 °C. After centrifugation, take 600 µL of supernatant, drain it in a freeze vacuum concentrator, and then add 600 µL of the complex solution (methanol: H2O = 1:9, v:v) for reconstitution. Vortex the mixture for 1 min, ultrasonicate it for 10 min in a 4 °C water bath, and then centrifuge it at 25,000 rpm for 15 min at 4 °C. The supernatant is then placed in a loading bottle. The separation and detection of metabolites were performed using the Waters2D UPLC tandem Q Exactive HF high-resolution mass spectrometer. For detailed statistical tests performed during microbiome analysis, refer to the works of Palevich et al [37].

### Comprehensive analysis of microbiome and metabolomic

Based on the abundance of metabolites and relative abundance of microorganisms, we conducted joint analysis of fecal microbiome and metabolome, mainly using Spearman correlation analysis, CCA analysis, etc. Through joint analysis, we obtained the chord diagram of the correlation between metabolic pathways and microbial taxa and the heat map of the correlation between differential metabolites and microbial taxa.

### Statistical analysis

SPSS 26.0 statistical software was used to analyse the data, and the mean ± SEM. was used to express the experimental data; the data were analysed by t-test for difference; *p* < 0.05 indicated that the difference was statistically significant.

### Electronic supplementary material

Below is the link to the electronic supplementary material.

**Additional file 1**: Samples Quality Control. (Docx 15kb)

**Additional file 2**: Statistical analysis of differential metabolites. (Docx 14kb)

**Additional file 3**: Top 10 different metabolites in PT group and NC group. (Docx 18kb)

**Additional file 4**: Results of fecal metabolite enrichment pathway in PT group and NC group. (Docx 16kb)

**Additional file 5**: Enrichment results of fecal metabolite metabolism pathways of goats in PT and NC groups. (Docx 16kb)

**Additional file 6**: Steroid hormone biosynthesis (positive ion model). (Docx 48kb)

**Additional file 7**: Bile secretion (positive ion model). (Docx 50kb)

**Additional file 8**: Bile secretion (Negative ion mode). (Docx 50kb)

**Additional file 9**: Phenylalanine, tyrosine and tryptophan biosynthesis (Negative ion mode). (Docx 34kb)

**Additional file 10**: Composition of feed. (Docx 16kb)

**Additional file 11**: The composition of feed nutrients. (Docx 17kb)

## Data Availability

We have submitted the fecal microbiome sequencing data to the SRA database in NCBI under with accession numbers: PRJNA946138 and the name is “pregnancy toxaemia goat fecal microbiome sequencing”. Metabolomics data have been uploaded to MetaboLights Website, and the accession numbers is MTBLS7519.

## Abbreviations

PT: Pregnancy toxemia
NC: Negative control
ALT: Alanine aminotransferase
AST: Aspartate aminotransferase
ALP: Alkaline phosphatase
GGT: Glutamyl transpeptidase
TP: Total protein
ALB: Albumin
GLB: Globulin
A/G: Albumin /globulin
TBIL-V: Total Bilirubin
GLU: Glucose
Ca: Calcium
P: Phosphorus
TC: Total cholesterol
TG: Triglyceride
CK: Creatine kinase
LDH: Lactate dehydrogenase
FUN: Fructosamine
B-HB: β-hydroxybutyric acid
OTUs: Operational taxonomic units
