# Supplementary material for: Analysis of fecal microbiome and metabolome changes in goats with pregnant toxemia
Source: BMC Vet Res. 2024 Jan 3;20:2. doi: 10.1186/s12917-023-03849-0 (PMC10763682; doi:10.1186/s12917-023-03849-0)
Supplement: Supplementary file 1 — Additional file 1: Samples Quality Control. (Docx 15kb) [file 12917_2023_3849_MOESM1_ESM.docx]

**Additional file 1**

**Statistics of the number of compounds under positive ion mode and negative ion mode**

| Mode | Total ions | High mass ion number | Percentage (%) |
| --- | --- | --- | --- |
| pos | 5674 | 4901 | 86.38 |
| neg | 2369 | 2148 | 90.67 |

**Parameters of PLS-DA model**

| Mode | Group | A | R2Y(cum) | Q2(cum) | R2 | Q2 |
| --- | --- | --- | --- | --- | --- | --- |
| pos | PT-NC | 3 | 0.99 | 0.78 | (0.0,0.95) | (0.0,﹣0.72) |
| neg | PT-NC | 3 | 0.99 | 0.75 | （0.0,0.94） | （0.0,﹣0.77） |
