# Supplementary material for: Analysis of fecal microbiome and metabolome changes in goats with pregnant toxemia
Source: BMC Vet Res. 2024 Jan 3;20:2. doi: 10.1186/s12917-023-03849-0 (PMC10763682; doi:10.1186/s12917-023-03849-0)
Supplement: Supplementary file 2 — Additional file 2: Statistical analysis of differential metabolites. (Docx 14kb) [file 12917_2023_3849_MOESM2_ESM.docx]

**Additional file 6**

**
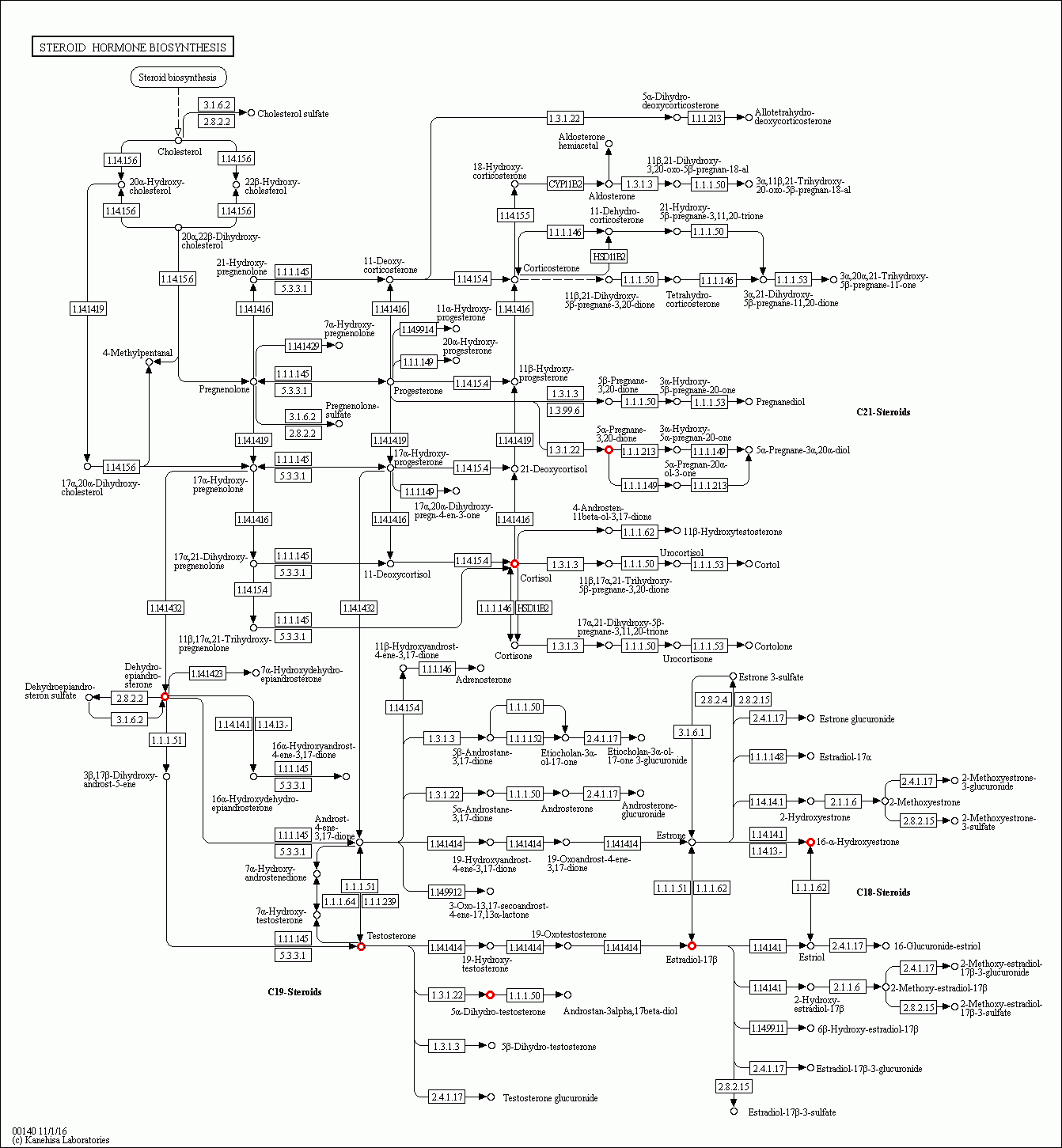
 Steroid hormone biosynthesis (positive ion model)**

Note: Small boxes: enzymes; small circles: metabolites (red indicates that the metabolite is a differential metabolite and is up-regulated in the comparison group, blue indicates that the metabolite is a differential metabolite and is down-regulated in the comparison group); arrow: reaction direction; Large box: other metabolic pathways.
