# Supplementary material for: Analysis of fecal microbiome and metabolome changes in goats with pregnant toxemia
Source: BMC Vet Res. 2024 Jan 3;20:2. doi: 10.1186/s12917-023-03849-0 (PMC10763682; doi:10.1186/s12917-023-03849-0)
Supplement: Supplementary file 3 — Additional file 3: Top 10 different metabolites in PT group and NC group. (Docx 18kb) [file 12917_2023_3849_MOESM3_ESM.docx]

**Additional file 9**

**
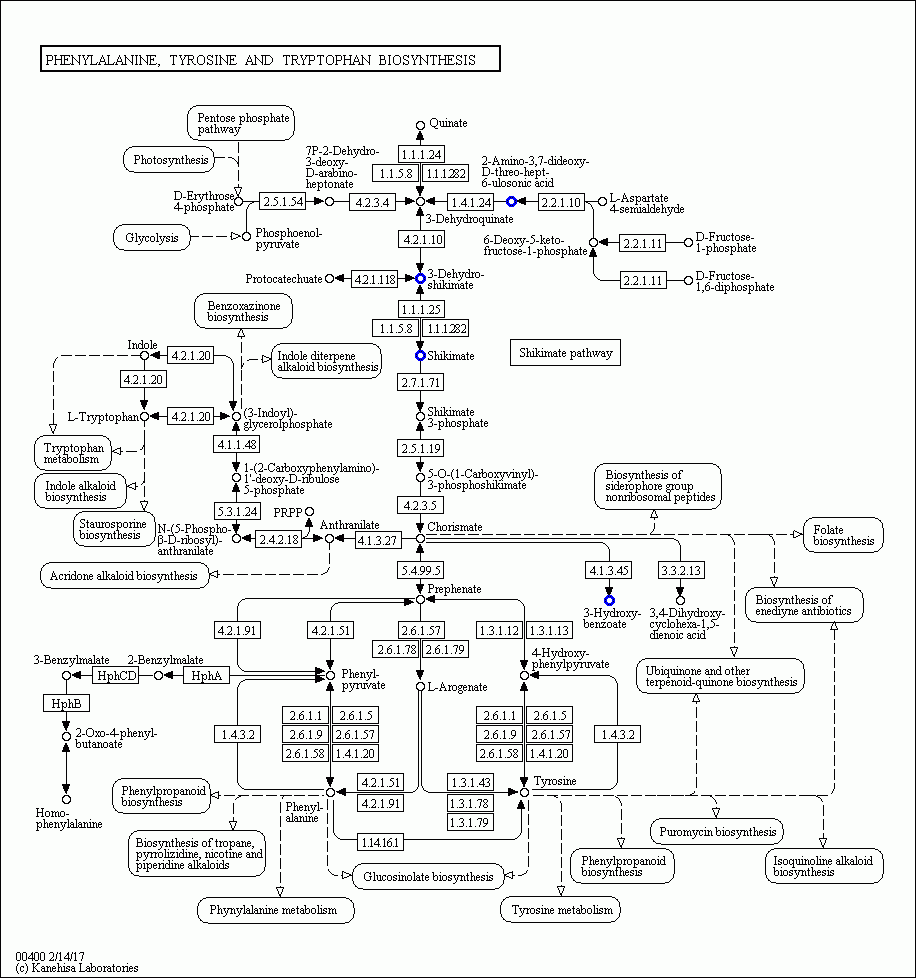
Phenylalanine, tyrosine and tryptophan biosynthesis (Negative ion mode)**

Note: Small boxes: enzymes; small circles: metabolites (red indicates that the metabolite is a differential metabolite and is up-regulated in the comparison group, blue indicates that the metabolite is a differential metabolite and is down-regulated in the comparison group); arrow: reaction direction; Large box: other metabolic pathways.
