# Supplementary material for: Analysis of fecal microbiome and metabolome changes in goats with pregnant toxemia
Source: BMC Vet Res. 2024 Jan 3;20:2. doi: 10.1186/s12917-023-03849-0 (PMC10763682; doi:10.1186/s12917-023-03849-0)
Supplement: Supplementary file 4 — Additional file 4: Results of fecal metabolite enrichment pathway in PT group and NC group. (Docx 16kb) [file 12917_2023_3849_MOESM4_ESM.docx]

**Additional file 2**

**Statistical analysis of differential metabolites in feces of PT group and NC group**

| Mode | Group | Differential metabolites total number | Upregulated | Downregulated |
| --- | --- | --- | --- | --- |
| pos | PT-NC | 125 | 89 | 36 |
| neg | PT-NC | 100 | 51 | 49 |
