# Supplementary material for: Analysis of fecal microbiome and metabolome changes in goats with pregnant toxemia
Source: BMC Vet Res. 2024 Jan 3;20:2. doi: 10.1186/s12917-023-03849-0 (PMC10763682; doi:10.1186/s12917-023-03849-0)
Supplement: Supplementary file 5 — Additional file 5: Enrichment results of fecal metabolite metabolism pathways of goats in PT and NC groups. (Docx 16kb) [file 12917_2023_3849_MOESM5_ESM.docx]

**Additional file 8**

**
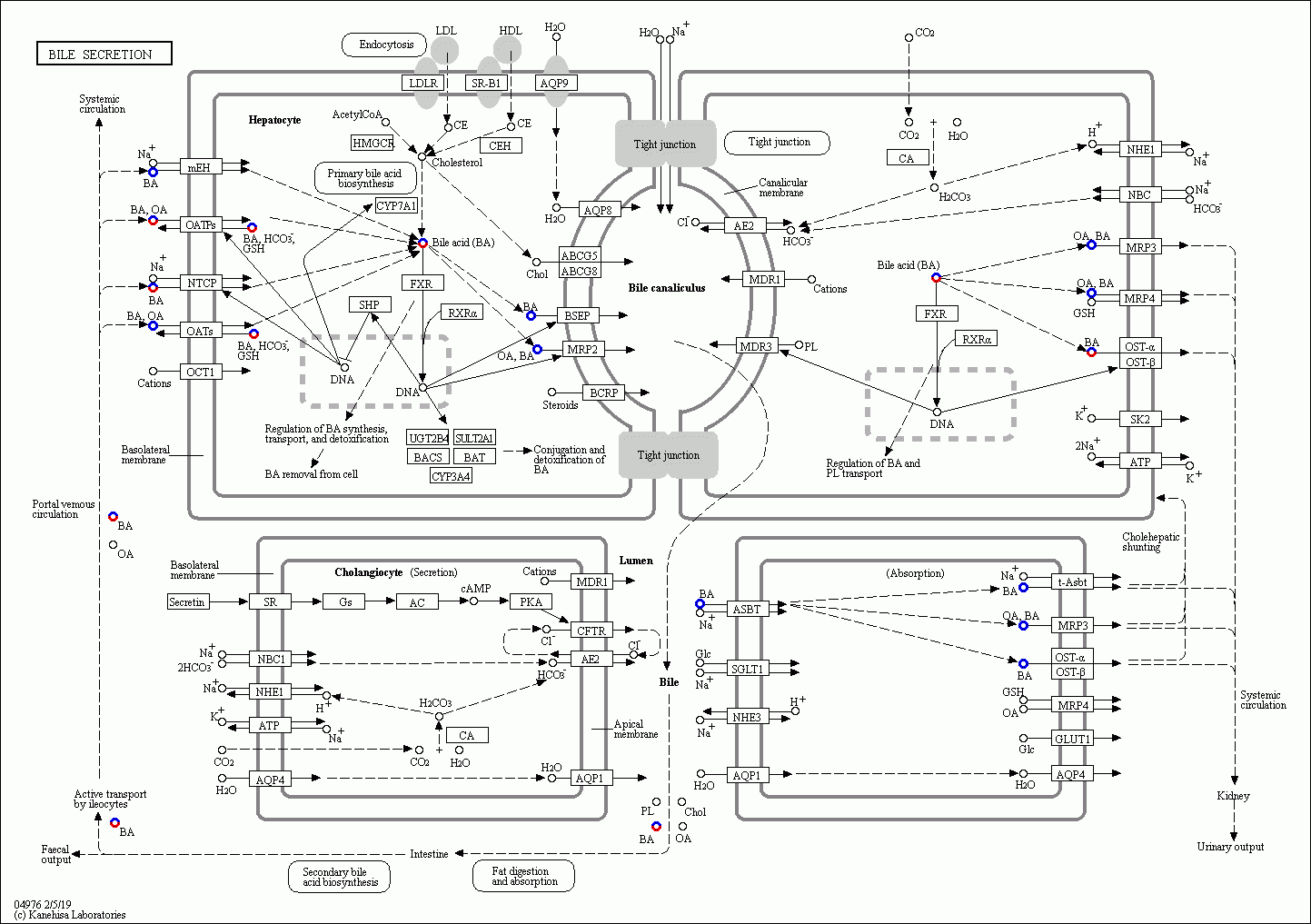
Bile secretion (Negative ion mode)**

Note: Small boxes: enzymes; small circles: metabolites (red indicates that the metabolite is a differential metabolite and is up-regulated in the comparison group, blue indicates that the metabolite is a differential metabolite and is down-regulated in the comparison group); arrow: reaction direction; Large box: other metabolic pathways.
