# Supplementary material for: Analysis of fecal microbiome and metabolome changes in goats with pregnant toxemia
Source: BMC Vet Res. 2024 Jan 3;20:2. doi: 10.1186/s12917-023-03849-0 (PMC10763682; doi:10.1186/s12917-023-03849-0)
Supplement: Supplementary file 6 — Additional file 6: Steroid hormone biosynthesis (positive ion model). (Docx 48kb) [file 12917_2023_3849_MOESM6_ESM.docx]

**Additional file 4**

**Comparison table of differential metabolites and metabolic pathways in feces of goats in PT group and NC group (positive ion mode)**

| Metabolic pathways | Upregulate differential etabolites | Downregulate  differential metabolites |
| --- | --- | --- |
| Steroid hormone biosynthesis | 6α-hydroxyestrone；Cortisol；Dehydroepiandrosterone；Β-estradiol；Testosterone；5α-dihydrotestosterone；5α-pregnan-3,20-dione | / |
| Ovarian steroidogenesis | Dehydroepiandrosterone；Β-estradiol；Testosterone | / |
| Bile secretion | Thromboxane b2；Cortisol；Glycocholate；Deoxycholate | Glycocholate |
| Serotonergic synapse | Thromboxane b2；11-dehydro thromboxane b2 | / |
| Aldosterone-regulated sodium reabsorption | Cortisol | / |
| Endocrine and other factor-regulated calcium reabsorption | Β-estradiol | / |
| Estrogen signaling pathway | Β-estradiol | / |
| Arachidonic acid metabolism | Thromboxane b2；11-dehydro thromboxane b2 | / |
| Cholesterol metabolism | Glycocholate | Glycocholate |
| Tyrosine metabolism | / | 3,4-dihydroxyphenylglycol；Mhpg |
| Prolactin signaling pathway | Β-estradiol | / |
| Cortisol synthesis and secretion | Cortisol | / |

**Comparison table of differential metabolites and metabolic pathways in feces of goats in PT group and NC group (negative ion mode)**

| Metabolic pathways | Upregulate differential metabolites | Downregulate differential metabolites |
| --- | --- | --- |
| Phenylalanine, tyrosine and tryptophan biosynthesis | / | 3-dehydroshikimic acid；2-amino-2,3,7-trideoxy-d-lyxo-hept-6-ulosonic acid；Shikimate；3-hydroxybenzoic acid |
| Bile secretion | Chenodeoxycholate；Deoxycholic acid；Lithocholic acid | Glycocholic acid |
| Tyrosine metabolism | / | Fumarylacetoacetic acid；(2z,4e)-5-hydroxy-2,4-pentadiene-1,2,5-tricarboxylic acid；2,5-dihydroxybenzaldehyde |
| Primary bile acid biosynthesis | Chenodeoxycholate | Glycocholic acid |
| Endocrine and other factor-regulated calcium reabsorption | Calcitriol | / |
| Parathyroid hormone synthesis, secretion and action | Calcitriol | / |
| Cholesterol metabolism | / | Glycocholic acid |
