# Supplementary material for: Analysis of fecal microbiome and metabolome changes in goats with pregnant toxemia
Source: BMC Vet Res. 2024 Jan 3;20:2. doi: 10.1186/s12917-023-03849-0 (PMC10763682; doi:10.1186/s12917-023-03849-0)
Supplement: Supplementary file 8 — Additional file 8: Bile secretion (Negative ion mode). (Docx 50kb) [file 12917_2023_3849_MOESM8_ESM.docx]

**Additional file 3**

**Top 10 up-regulated fecal differential metabolites in PT and NC groups (Positive ion mode)**

| Differential metabolite name | VIP | Fold-change | q- value |
| --- | --- | --- | --- |
| (9alpha,13beta)-2-oxospartein-13-yl (2e)-2-methyl-2-butenoate | 3.0165 | 210.4678 | 0.0027 |
| Calpeptin | 2.2187 | 34.4584 | 0.0296 |
| 1-o-palmitoyl-2-o-arachidonoyl-sn-glycero-3-phosphocholine | 2.1652 | 24.5018 | 0.0062 |
| 20-trifluoromethyl-leukotriene b4 | 2.6126 | 21.2591 | 0.0001 |
| 11-dehydro thromboxane b2 | 2.5513 | 19.7032 | 0.0004 |
| Cyclohexanone | 2.1404 | 19.3739 | 0.0259 |
| Mitragynine | 2.0465 | 18.7896 | 0.0414 |
| Di-n-amyl phthalate | 2.5921 | 16.6919 | 0 |
| Remifentanil | 1.9592 | 16.3782 | 0.0415 |
| Estrone | 2.2371 | 16.3376 | 0.0067 |

**Top 10 fecal differential metabolites down-regulated in PT and NC groups (Positive ion mode)**

| Differential metabolite name | VIP | Fold-change | q-value |
| --- | --- | --- | --- |
| Daphnoretin | 1.8104 | 0.0109 | 0.0363 |
| Dihydromethylsterigmatocystin | 1.8143 | 0.0126 | 0.0462 |
| 5,7-dihydroxy-2-(3-hydroxy-4-methoxyphenyl)-3,6-dimethoxy-2,3-dihydro-4h-chromen-4-one | 1.8407 | 0.0127 | 0.038 |
| Bicoumol | 1.8779 | 0.019 | 0.0289 |
| Gemifloxacin | 1.7335 | 0.0385 | 0.0309 |
| O-succinylbenzoate | 1.7636 | 0.0478 | 0.0184 |
| Scoparone | 2.4241 | 0.0624 | 0.0029 |
| Urolithin b | 2.3611 | 0.0667 | 0.0089 |
| Magnoline | 1.5637 | 0.0735 | 0.0325 |
| Fructoselysine | 2.0654 | 0.0751 | 0.0004 |

**Additional file 3**

**Top 10 up-regulated fecal differential metabolites in PT and NC group (Negative ion mode)**

| Differential metabolite name | VIP | Fold-change | q-value |
| --- | --- | --- | --- |
| (9alpha,13beta)-2-oxospartein-13-yl (2e)-2-methyl-2-butenoate | 2.6248 | 252.9077 | 0.0056 |
| Chenodeoxycholate | 2.6489 | 117.8677 | 0.0009 |
| 1-o-palmitoyl-2-o-arachidonoyl-sn-glycero-3-phosphocholine | 2.6499 | 75.0033 | 0.0004 |
| Marbofloxacin | 2.2643 | 53.2107 | 0.0016 |
| Cambendazole | 2.0683 | 36.703 | 0.0133 |
| (10s)-juvenile hormone iii diol | 1.8829 | 28.6192 | 0.0259 |
| Alfuzosin | 2.4499 | 23.3881 | 0 |
| Vibegron | 2.1592 | 20.3249 | 0.0071 |
| 8-isoprostaglandin f1β | 2.1866 | 17.4121 | 0.0002 |
| Limonin glucoside | 1.5249 | 17.0701 | 0.023 |

**Top 10 fecal differential metabolites down-regulated in PT and NC groups (Negative ion mode)**

| Differential metabolite name | VIP | Fold-change | q-value |
| --- | --- | --- | --- |
| Dihydromethylsterigmatocystin | 2.2475 | 0.0006 | 0.0162 |
| Daphnoretin | 2.2025 | 0.0007 | 0.0108 |
| Bicoumol | 2.1657 | 0.0013 | 0.0183 |
| 7-carboxy-7-deazaguanine | 1.3502 | 0.0073 | 0.047 |
| Adlupone | 1.7812 | 0.0087 | 0.0149 |
| 4-hydroxycoumarin | 1.9352 | 0.0099 | 0.0128 |
| Semilicoisoflavone b | 1.7073 | 0.0516 | 0.0191 |
| Urolithin b | 2.2455 | 0.0554 | 0.0057 |
| Artocarpin | 1.5335 | 0.0598 | 0.0128 |
| 3-methyl-2-butenyl caffeate | 1.9633 | 0.0625 | 0.0004 |
