# Supplementary material for: Analysis of fecal microbiome and metabolome changes in goats with pregnant toxemia
Source: BMC Vet Res. 2024 Jan 3;20:2. doi: 10.1186/s12917-023-03849-0 (PMC10763682; doi:10.1186/s12917-023-03849-0)
Supplement: Supplementary file 9 — Additional file 9: Phenylalanine, tyrosine and tryptophan biosynthesis (Negative ion mode). (Docx 34kb) [file 12917_2023_3849_MOESM9_ESM.docx]

**Additional file 5**

**Enrichment results of fecal metabolite metabolism pathways of goats in PT and NC groups (Positive ion mode)**

| Metabolic pathways | Quantity | Total | p-value | Pathway Number |
| --- | --- | --- | --- | --- |
| Steroid hormone biosynthesis | 7 | 99 | 1.6474E-07 | map00140 |
| Ovarian steroidogenesis | 3 | 24 | 0.00012701 | map04913 |
| Bile secretion | 4 | 97 | 0.00068442 | map04976 |
| Metabolic pathways | 16 | 1706 | 0.00134176 | map01100 |
| Serotonergic synapse | 2 | 42 | 0.01291392 | map04726 |
| Aldosterone-regulated sodium reabsorption | 1 | 8 | 0.03242642 | map04960 |
| Endocrine and other factor-regulated calcium reabsorption | 1 | 8 | 0.03242642 | map04961 |
| Estrogen signaling pathway | 1 | 8 | 0.03242642 | map04915 |
| Arachidonic acid metabolism | 2 | 75 | 0.03816468 | map00590 |
| Cholesterol metabolism | 1 | 10 | 0.04036952 | map04979 |
| Tyrosine metabolism | 2 | 78 | 0.04097793 | map00350 |
| Prolactin signaling pathway | 1 | 11 | 0.04431691 | map04917 |
| Cortisol synthesis and secretion | 1 | 12 | 0.04824827 | map04927 |

**Table of enrichment results of fecal metabolite metabolism pathways of goats in PT and NC groups（Negative ion mode）**

| Metabolic pathways | Quantity | Total | *P*-value | Pathway Number |
| --- | --- | --- | --- | --- |
| Phenylalanine, tyrosine and tryptophan biosynthesis | 4 | 35 | 4.55039E-06 | map00400 |
| Bile secretion | 4 | 97 | 0.000259882 | map04976 |
| Tyrosine metabolism | 3 | 78 | 0.00198221 | map00350 |
| Primary bile acid biosynthesis | 2 | 47 | 0.009872301 | map00120 |
| Endocrine and other factor-regulated calcium reabsorption | 1 | 8 | 0.02525416 | map04961 |
| Parathyroid hormone synthesis, secretion and action | 1 | 10 | 0.03146909 | map04928 |
| Cholesterol metabolism | 1 | 10 | 0.03146909 | map04979 |
