# Supplementary material for: Analysis of fecal microbiome and metabolome changes in goats with pregnant toxemia
Source: BMC Vet Res. 2024 Jan 3;20:2. doi: 10.1186/s12917-023-03849-0 (PMC10763682; doi:10.1186/s12917-023-03849-0)
Supplement: Supplementary file 10 — Additional file 10: Composition of feed. (Docx 16kb) [file 12917_2023_3849_MOESM10_ESM.docx]

**Additional file 10**

**Composition of feed**

| Ingredient composition | Diets（%） |
| --- | --- |
| Maize | 36 |
| Concentrated material | 16 |
| Peanut shell | 17.5 |
| Corn husk | 17.5 |
| Grass flour | 12 |
| Dawei Wang (additive) | 0.2 |
| Salt | 0.3 |
| Sodium bicarbonate | 0.5 |
