# Supplementary material for: Analysis of fecal microbiome and metabolome changes in goats with pregnant toxemia
Source: BMC Vet Res. 2024 Jan 3;20:2. doi: 10.1186/s12917-023-03849-0 (PMC10763682; doi:10.1186/s12917-023-03849-0)
Supplement: Supplementary file 11 — Additional file 11: The composition of feed nutrients. (Docx 17kb) [file 12917_2023_3849_MOESM11_ESM.docx]

**Additional file 11**

**The composition of feed nutrients**

| Items | Diets |
| --- | --- |
| Metabolic energy (MJ/kg DM) | 12.45 |
| CP (% DM) | 14.83 |
| Crude fat (% DM) | 2.65 |
| NDF (% DM) | 43.58 |
| ADF (% DM) | 32.07 |
| Crude ash (% DM) | 6.54 |
| Calcium (% DM) | 0.55 |
| Phosphorus (% DM) | 0.47 |
